# Total disc replacement using tissue-engineered intervertebral discs in the canine cervical spine

**DOI:** 10.1371/journal.pone.0185716

**Published:** 2017-10-20

**Authors:** Yu Moriguchi, Jorge Mojica-Santiago, Peter Grunert, Brenton Pennicooke, Connor Berlin, Thamina Khair, Rodrigo Navarro-Ramirez, Rodolfo J. Ricart Arbona, Joseph Nguyen, Roger Härtl, Lawrence J. Bonassar

**Affiliations:** 1 Department of Neurological Surgery, Weill Cornell Medical College, New York, NY, United States of America; 2 Meinig School of Biomedical Engineering, Cornell University, Ithaca, NY, United States of America; 3 Center of Comparative Medicine and Pathology, Memorial Sloan Kettering Cancer Center & Weill Cornell Medicine, New York, NY, United States of America; 4 Healthcare Research Institute, Hospital for Special Surgery, Hospital for Special Surgery, New York, NY, United States of America; Universita degli Studi di Palermo, ITALY

## Abstract

The most common reason that adults in the United States see their physician is lower back or neck pain secondary to degenerative disc disease. To date, approaches to treat degenerative disc disease are confined to purely mechanical devices designed to either eliminate or enable flexibility of the diseased motion segment. Tissue engineered intervertebral discs (TE-IVDs) have been proposed as an alternative approach and have shown promise in replacing native IVD in the rodent tail spine. Here we demonstrate the efficacy of our TE-IVDs in the canine cervical spine. TE-IVD components were constructed using adult canine annulus fibrosis and nucleus pulposus cells seeded into collagen and alginate hydrogels, respectively. Seeded gels were formed into a single disc unit using molds designed from the geometry of the canine spine. Skeletally mature beagles underwent discectomy with whole IVD resection at levels between C3/4 and C6/7, and were then divided into two groups that received only discectomy or discectomy followed by implantation of TE-IVD. Stably implanted TE-IVDs demonstrated significant retention of disc height and physiological hydration compared to discectomy control. Both 4-week and 16-week histological assessments demonstrated chondrocytic cells surrounded by proteoglycan-rich matrices in the NP and by fibrocartilaginous matrices in the AF portions of implanted TE-IVDs. Integration into host tissue was confirmed over 16 weeks without any signs of immune reaction. Despite the significant biomechanical demands of the beagle cervical spine, our stably implanted TE-IVDs maintained their position, structure and hydration as well as disc height over 16 weeks *in vivo*.

## Introduction

Degenerative disc disease (DDD) is a prevalent clinical condition occurring in 40% of individuals younger than 30 and more than 90% of individuals over the age of 50 [1], which can lead to nerve compression and chronic back pain. Though pharmacological and physiotherapeutic treatments relieve early symptoms [2], surgical intervention is required in over half a million patients in the US annually [3]. The current surgical standard to treat DDD involves the removal of the entire IVD followed by fusion of the adjacent vertebrae or interposition of a mechanical disc prosthesis to preserve motion. However, fusion brings risks of possible pseudarthrosis and adjacent segment disease, resulting in a higher rate of reoperation in these patients [4, 5]. Prosthetic total disc replacement devices, developed to maintain segmental mobility, are an alternative to fusion surgery. However, recent studies have shown that total disc replacement also leads to adjacent segment disease [4, 6]. The main concern with current treatment options for DDD—conservative or surgical—is that they fail to treat the underlying etiology and the degenerated disc remains unrepaired.

**Fig 1:**
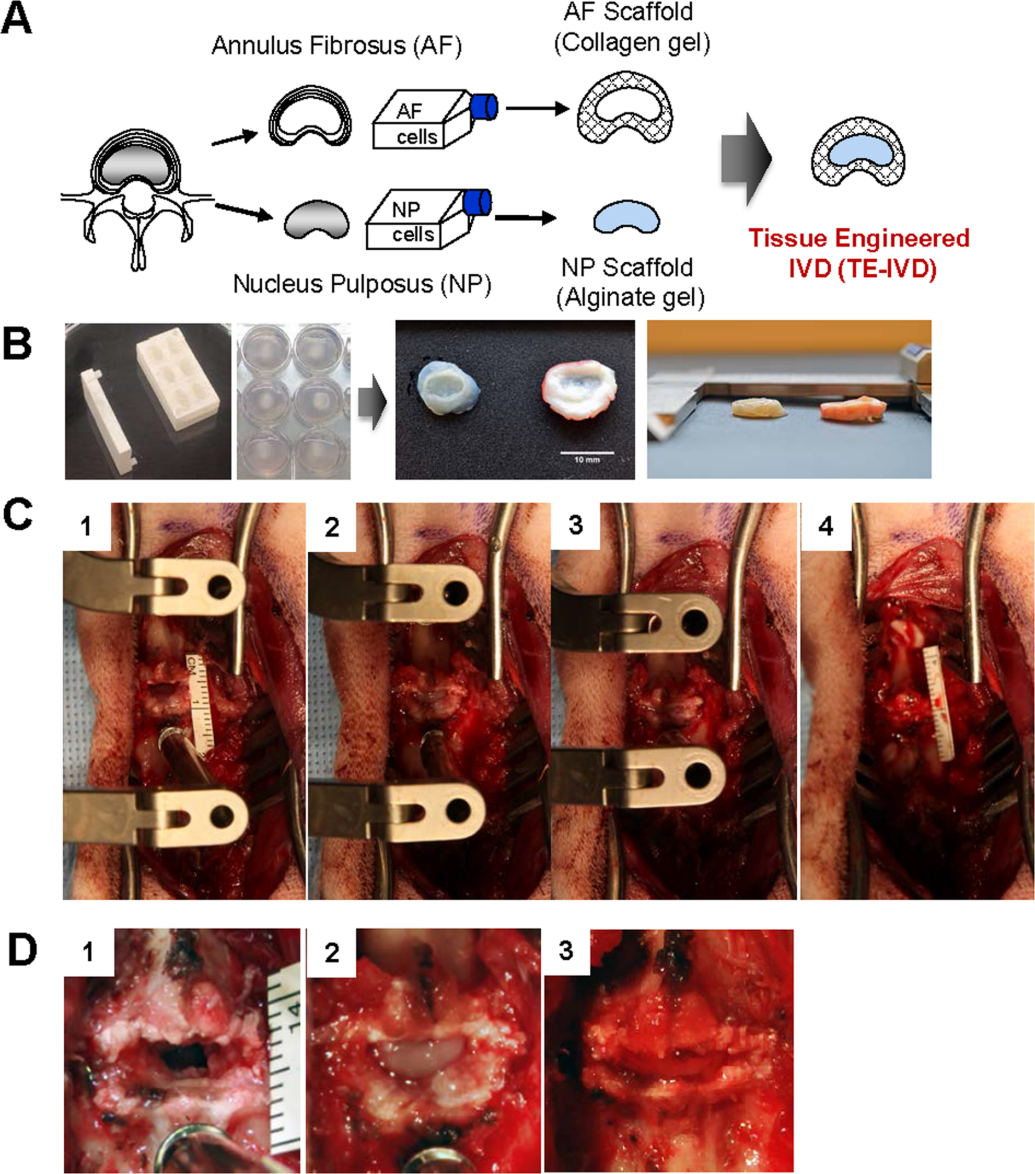
A) Schematic picture of disc tissue engineering. NP and AF cells were separately isolated from canine lumbar spine and cultured in vitro. Cultured NP and AF cells were seeded in alginate and collagen gels, respectively, and subsequently both composites were combined into a TE-IVD. B) Photographs of TE-IVD fabrication. Cultured NP cells were injected into a predesigned mold and encircled with two layers of AF cell-laden collagen gels. These AF layers circumferentially contract over cultivation time until they reach a size similar to native IVD as observed in top view and side view. C) Total discectomy and TE-IVD implantation were performed anteriorly under segmental distraction. D) Upon distraction release, stably transplanted TE IVDs remained in place and were secured in the disc space.

To overcome the limitations of available treatments and enhance patient care outcome, biological approaches to IVD repair or regeneration are of increasing interest. The first attempt to reconstruct a whole disc segment with biological implants now dates back approximately 10 years [7]. The feasibility of allogenic disc transplantation was demonstrated in clinical trials with favorable outcomes over a five-year follow-up. Despite the challenge of widespread deployment of this strategy due to limited implant availability and potential disease transmission, the results obtained were encouraging. Since Langer and Vacanti pioneered the multidisciplinary field of tissue engineering in 1993 [8], much effort has been directed towards the construction of functional substitutes for damaged disc tissues, especially for advanced stages of disc degeneration with extensive loss of extracellular matrix and functional structure. Numerous studies have assessed tissue-engineered whole disc constructs *in vitro*, but few have looked at these constructs *in vivo*. Mizuno et al. first developed the tissue engineered disc composed of NP cells seeded into an alginate hydrogel surrounded by a polyglycolic acid and polylactic acid scaffold seeded with AF cells. This *de novo* construct was implanted in the subcutaneous space of the dorsum of athymic mice, demonstrating the feasibility of creating a composite IVD with both AF and NP tissues [9, 10]. Several other studies have reported the development of composite tissue engineered IVD constructs, using combinations of materials such as demineralized bone matrix gelatin with type II collagen, hyaluronate and chondroitin-6-sulfate (C2/HyA-CS) [11], electrospun polycaprolactone and agarose [12]; and self-assembled NP cells seeded onto calcium polyphosphate [13]. We have shown previously the development of a tissue-engineered IVD composed of an inner NP cell-laden alginate core surrounded by an outer AF cell-laden collagen layer (Fig 1A) [14, 15]. We demonstrated the *in vivo* efficacy of this model at maintaining disc height and physiological hydration, when implanted in the rat tail for up to nine months [16–20].

Although these results are promising, there are several differences between the rat tail model and the human cervical spine that pose significant challenges for clinical translation [21–23]. The rat tail spine has a significantly different loading profile, and TE-IVDs will be exposed to higher axial loads when implanted into a human disc space. There are also anatomical differences, as the rat tail vertebrae lack a spinal canal containing nervous tissue as well as posterior bone and joint elements. To bring this innovation toward clinical application, we tested the feasibility of total disc replacement using TE IVDs in a beagle cervical spine model. We determined surgical conditions that promote stable implantation and investigated the ability of TE IVDs to maintain disc height, physiological hydration, and tissue viability in the present study.

## Results

### In vitro generation of tissue engineered-intervertebral discs (TE-IVDs)

We isolated nucleus pulpous (NP) cells and annulus fibrosus (AF) cells from the intervertebral discs from the cervical spines of skeletally mature beagles. These cells were separately cultured for 2–3 weeks and subsequently seeded in an alginate scaffold for NP and collagen for AF (Fig 1A and 1B). For TE-IVD construction, NP cell-laden alginate (25 × 10^6^ NP cells/mL) was injected into a predesigned mold and encircled with two layers of AF cell-laden collagen gels (1 × 10^6^ AF cells/mL). This 3D construct of TE-IVD demonstrated the contraction at the AF part during further two-week cultivation.

### Implanted TE-IVD maintained its position without neurological complication

Cervical discectomy was performed *in vivo* anteriorly as in human surgeries. The annulus fibrosus (AF) and nucleus pulposus (NP) were extensively resected under the microscope (Fig 1C and 1D). To determine a condition that promotes implant stability, we varied the surgical level operated on and presence or absence of ligament resection. With the lateral dimension over 80% of the disc width (most of the AF and all the NP portion), the entire antero-posterior depth of the disc was resected, confirming exposure of the posterior longitudinal ligament at the bottom (PLL). The PLL was resected in four dogs, but not in the others (to help determine favorable implant conditions, as aforementioned). Subsequently, all implants were successfully inserted into the discectomized segment under segmental distraction using a CASPAR distraction system. Upon distraction release, half of the TE-IVDs remained stable (n = 6), while the other half (n = 6) were considered displaced based on the extent of anterior protrusion (The summaries of implant stability immediately after removal of distraction are summarized in S1–S3 Tables). PLL resection did not have a significant effect on implant stability (p = 0.072). None of the dogs demonstrated segmental instability of the experimental level and none of the TE-IVDs demonstrated a propensity of posterior displacement intraoperatively. All animals were neurologically normal immediately after surgery and remained so for the duration of the experiments, with neurological assessments for abnormalities in gait, wheelbarrow assessment, limb hopping, and plantar reflex.

**Fig 2:**
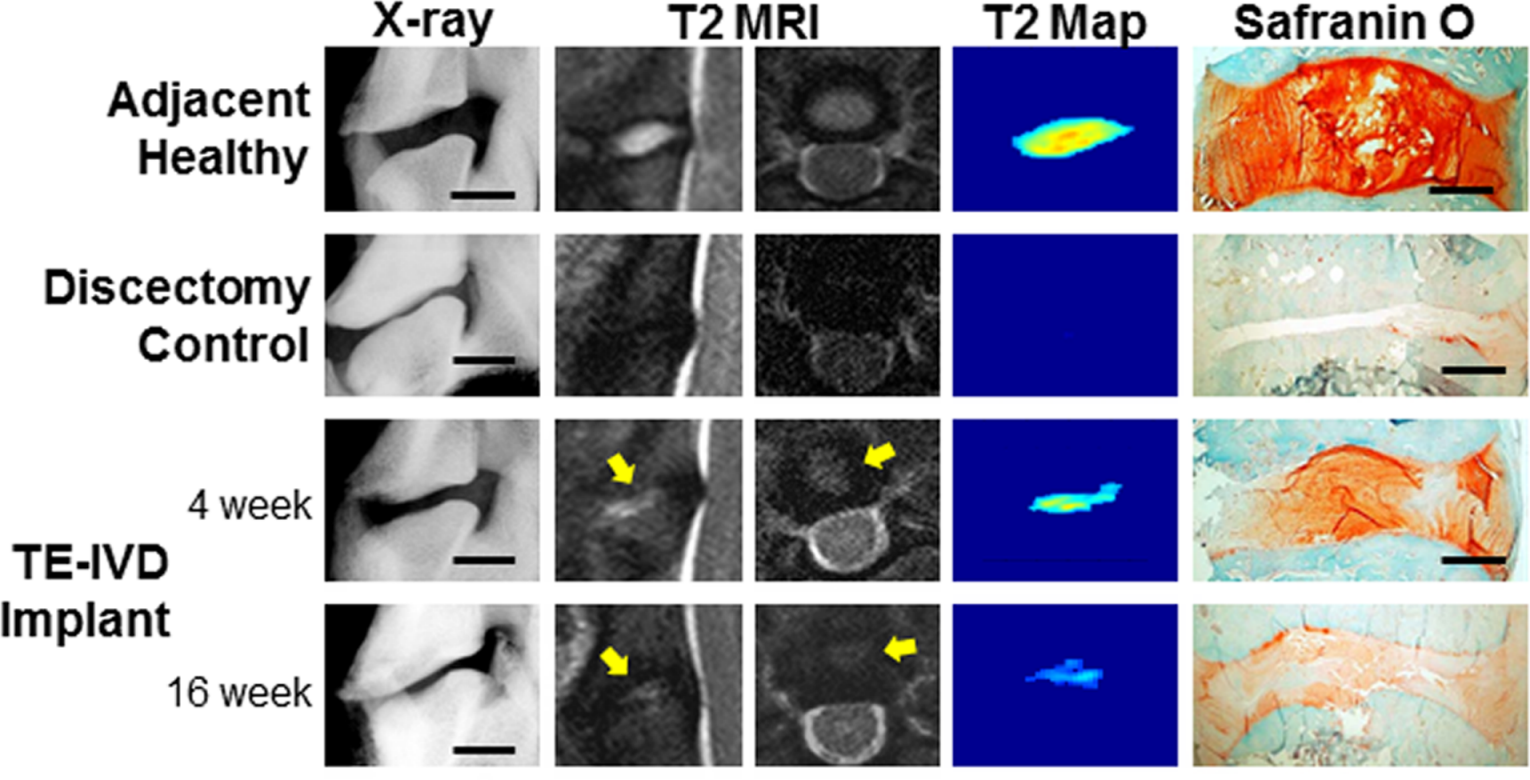
X-ray, MRI and histology of adjacent motion segment, discectomy, and TE-IVD at 4 and 16 weeks. Adjacent disc levels showed clear vertebral separation, strong hydration signal in sagittal and axial T2 MRI, highly localized T2 map signal, and abundant staining with Safranin O. Discectomy levels showed no vertebral separation and minimal T2 signal and no soft tissue present between vertebrae. Animals receiving stably transplanted TE-IVD showed clear vertebral separation, with tissue hydration noted in both sagittal and axial T2-weighed MRI (yellow arrows). At both 4 and 16 weeks after transplantation, proteoglycan-rich tissue was observed to be well integrated into the surrounding vertebrae.

All animals were imaged postoperatively using X-ray and MRI to monitor the implanted TE-IVD and screen for any host reaction (Fig 2). None of the dogs had neurological symptoms or adverse effects due to TE-IVD implantation, despite being free of external fixation or orthosis immediately after surgery. X-rays demonstrated no significant pathological abnormality observed in the vertebrae or signs of spinal malalignment such as spondylolisthesis among the groups. In MRIs, proximal adjacent discs served as a healthy control. The solely discectomized segments demonstrated a collapsed black disc on postoperative images and paucity of reparative tissues in the disc histology, suggesting that our discectomy procedure induced an incurable lesion by resecting the vast majority of the disc. In the TE-IVD implanted groups, location of the implant was confirmed by sagittal and axial MRIs. Stably implanted TE-IVDs demonstrated hyperintensity in T2-weighed images at 4 weeks, and maintained their position in the disc space with relatively decreased T2 intensity at 16 weeks. This loss in T2 signal is likely associated with a decrease in tissue hydration, and these data were consistent with the Safranin-O histology for proteoglycans, which are known to attract water into these tissues. Such histology also indicated a continuous connection between newly developed tissue and the surrounding vertebral body. This indicated that the implanted TE-IVDs engrafted within the disc space despite the biomechanical forces of the beagle cervical spine.

### Engrafted TE-IVDs produced functional tissue that maintained disc height and tissue hydration

**Fig 3:**
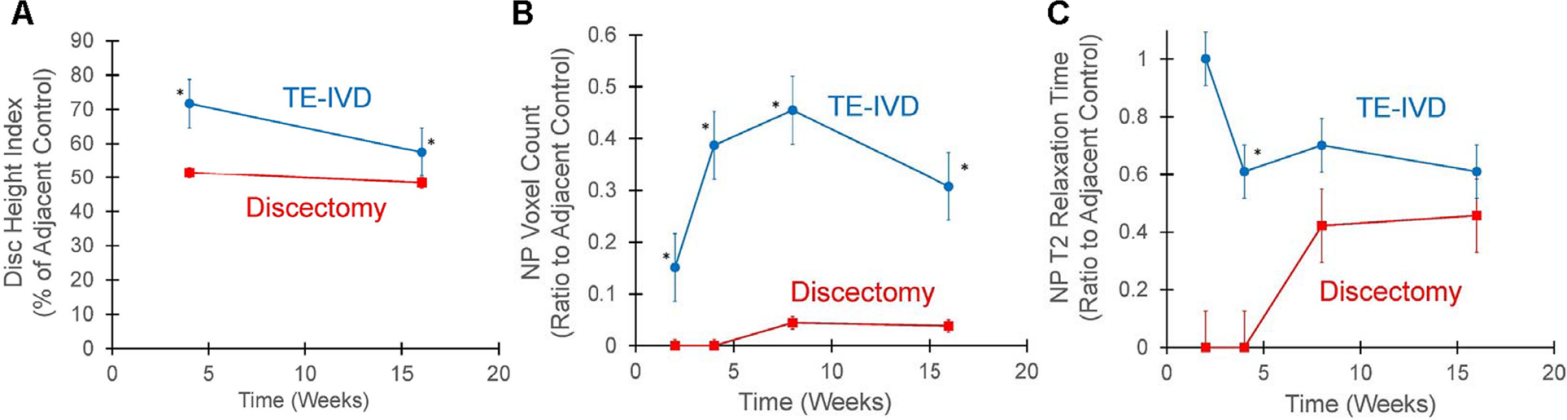
Quantitative analysis of disc height index and MRI. A) Stably implanted TE-IVD had significant retention of disc height compared to the discectomy control at 4 and 16 weeks (asterisks, p<0.001 at 4 and p = 0.012 at 16 weeks, respectively). B) TE-IVDs demonstrated significantly higher NP voxel counts than the discectomy controls after 4, 8, and 16 weeks (asterisks denote p<0.02 for all time points) C) TE-IVD had significantly higher T2-relaxation times than the discectomy group at 4 weeks (asterisk, p = 0.007). All data are represented as mean +/- standard error.

We investigated the ability of TE-IVDs to maintain disc height by measuring the disc height index as described previously [16]. At 4 weeks, the disc height indices of stably implanted TE-IVDs and discectomized discs were 71% and 51% of healthy control discs, respectively. Animals receiving TE-IVDs demonstrated significant decrease in disc height over time (p<0.001), with stable implants having larger disc height compared to the discectomy group at both 4 and 16 weeks (p<0.001 and p = 0.012, respectively) (Fig 3). We further assessed the size and hydration of the NP portion in the implanted TE-IVDs following an algorithm based on T2 relaxation time (T2-RT) measurements [24]. After 8 weeks, stable TE-IVDs had significantly higher NP voxel count than discectomy (p = 0.015) and demonstrated a significant increase in NP voxel count from 2 to 8 and 16 weeks (p = 0.009 and 0.020).

Mean NP T2-RT, a value representing NP hydration, was significantly higher for TE-IVDs than discectomy group at 2 and 4 weeks (p<0.001). There was a significant decrease in T2-RT between 2 and 4 weeks in the TE-IVD group, and only this group maintained physiological hydration of the NP of ~60% of adjacent healthy discs. Conversely, the discectomized segments did not show any region of T2 high intensity at 2 and 4 weeks, which confirmed that total discectomy was thoroughly performed. However, a very small amount of T2 high intensity region emerged in the discectomized segment after 8 weeks, probably due to the fluid accumulation induced by a secondary disc degenerative process. This pseudo-NP region observed in one specimen was responsible for paradoxical increase of the mean T2-RT at 8 and 16 weeks.

### TE-IVDs produced collagen- and proteoglycan-rich extracellular matrix in the AF and NP

**Fig 4:**
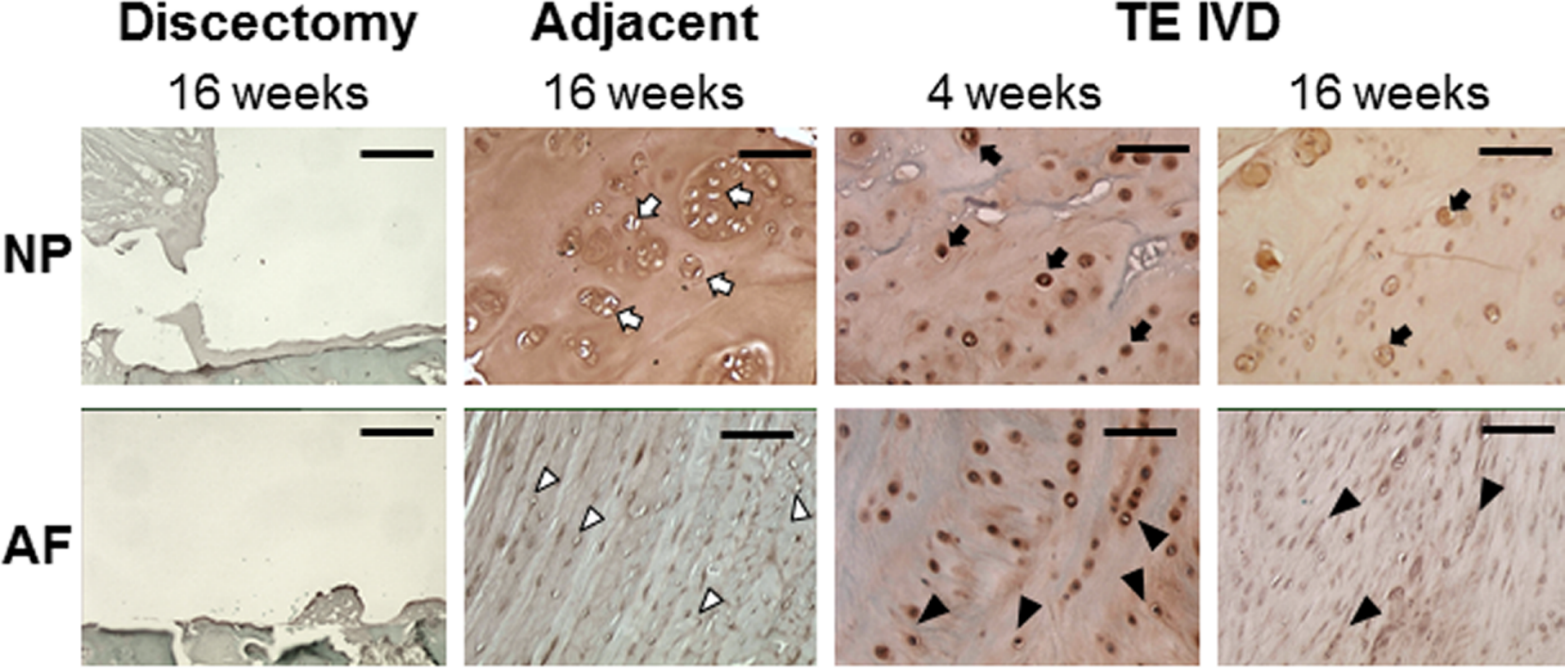
Safranin O staining showed an absence of tissue in the intervertebral space of samples in the discectomy group. Healthy tissue in the adjacent motion segment showed strong proteoglycan staining the NP, with numerous clusters of round chondrocytic cells (open arrows) and less staining in the AF, with more elongated fibrochondrocytes arranged in distinct fibrous lamellae (open arrow heads). At 4 weeks, TE-IVD samples had strong proteoglycan staining in both NP and AF, with rounded cells apparent in the NP (filled arrows) and more elongated cells arranged primitive lamellae in the AF. By 16 weeks, staining was more evident in the NP than AF, with some clustering of rounded chondrocytic cells (filled arrows). AF cells were clearly elongated (closed arrow heads) and aligned. All scale bars are 100 μm.

**Fig 5:**
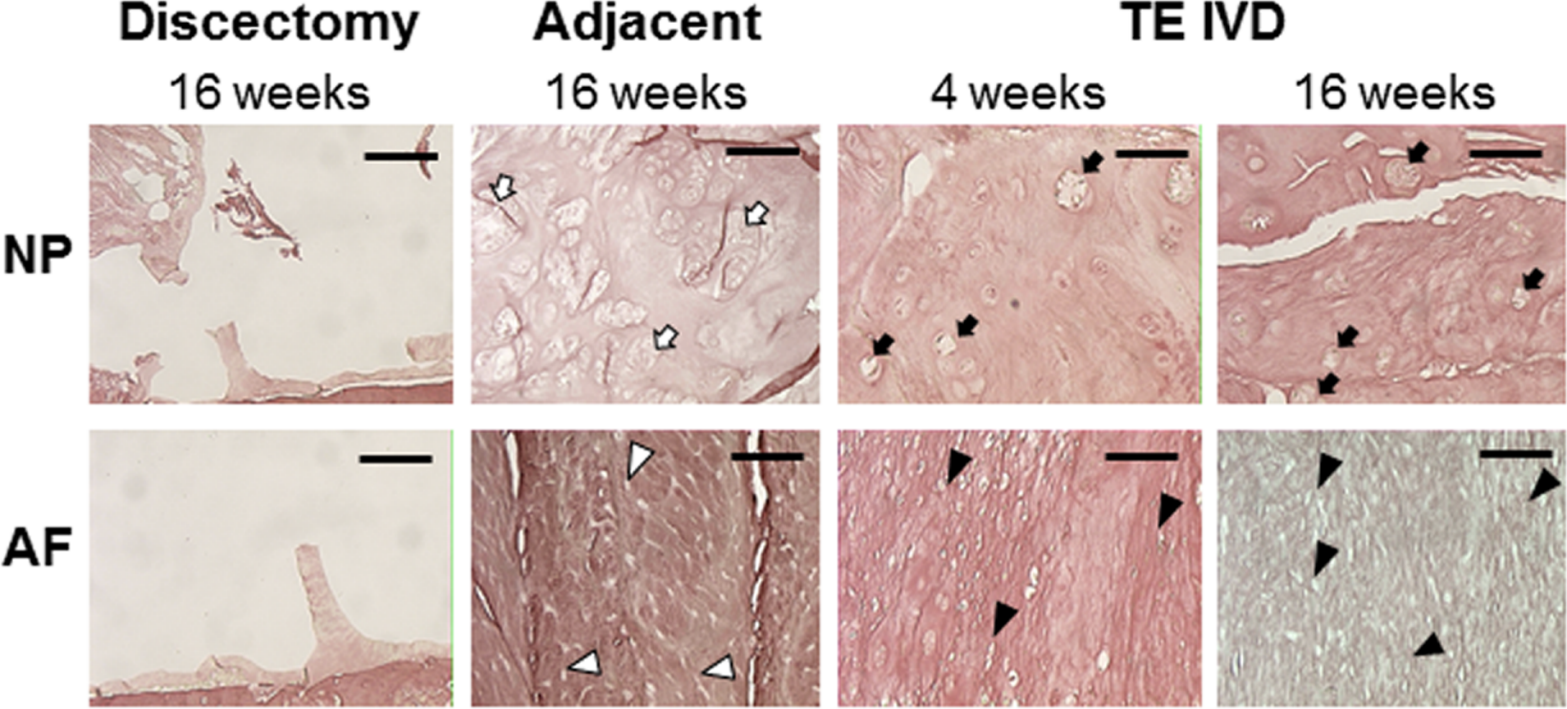
Picrosirius red staining showed an absence of tissue in the intervertebral space of samples in the discectomy group. Healthy tissue in the adjacent motion segment showed little staining for collagen in the NP with numerous clusters of round chondrocytic cells (open arrows) and strong staining in the AF, with more elongated fibrochondrocytes arranged in distinct fibrous lamellae (open arrow heads). At 4 weeks, TE-IVD samples had light collagen staining in the NP and stronger staining in the AF, with rounded cells apparent in the NP (filled arrows) and more elongated cells arranged primitive lamellae in the AF. By 16 weeks, staining was present in both NP than AF, with some clustering of rounded chondrocytic cells (filled arrows) in the NP and elongated cells in the AF (closed arrow heads). All scale bars are 100 μm.

A critical benchmark of success for a TE-IVD is the assessment of the biological function of the transplanted tissue as assessed by the ability to form robust extracellular matrix when implanted into the spine. To evaluate this biological function, we performed Safranin O staining for proteoglycans (Fig 4) and Picrosirius red staining for fibrillar collagen (Fig 5). Both stains demonstrated the successful removal of the IVD and the absence of any robust healing response from the discectomy group. In contrast, TE-IVD implants yielded proteoglycan-rich tissue with distinct morphological features of NP and AF. The morphologies of the NP and AF regions were more similar at 4 weeks, but highly distinct by 16 weeks. The central NP region contained rounded cells and cell clusters characteristic of a chondrocytic phenotype. The morphology of the NP was similar at 4 and 16 weeks, with the cell clustering that is characteristic of native NP more evident at 16 weeks. AF tissue showed a moderate level of proteoglycan staining at 4 weeks, with staining less pronounced at 16 weeks. At 4 weeks, cells were somewhat elongated, but aligned in nascent lamellae. By 16 weeks, cells were highly elongated, as in the native AF, and aligned into mature lamellae surrounding the NP. Notably, at either 4 or 16 weeks, there was little chronic inflammation or foreign body response to TE IVD implants evident from Safranin O (Fig 4), Picrosirius red (Fig 5), or Hematoxylin and eosin (S3 Fig) stains. At both time points, cells in both the AF and NP regions appeared healthy and there was no sign of implant rejection, despite the implants being seeded with allogenic cells.

### TE-IVDs integrated with neighboring vertebrae and reproduced a native disc shape and composite structure over 16 weeks of implantation

**Fig 6:**
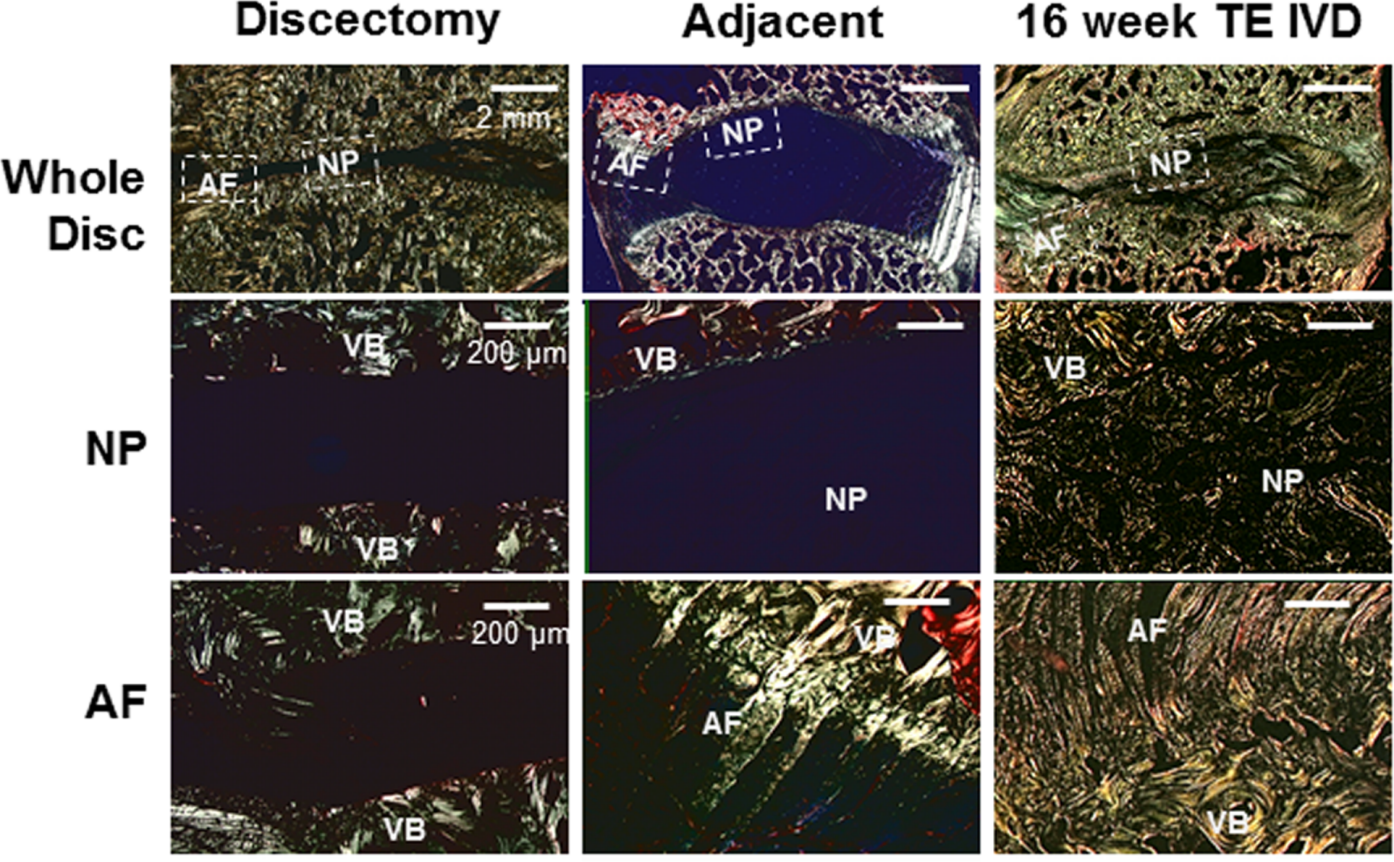
Picrosirius red-stained histology under polarized light showed birefringent features associated with collagen fibers. Discectomy samples show collagen organization in vertebrae, with no tissue in the intervertebral space. Adjacent motion segments show the absence of collagen fibers in the NP and large collagen fibers (~50–100 μm) inserting from the AF to the vertebral body (VB). At 16 weeks, TE-IVD samples show the presence of some small, unorganized collagen fibers in the NP, with larger (~20–50 μm) fibers that insert into the vertebral body (VB).

A major challenge in a tissue engineering approach to IVD repair is the integration of a newly grown implant into the surrounding vertebrae. To assess the integration of the implanted TE-IVD, we used polarized light microscopy in conjunction with Picrosirius red staining to evaluate the collagen organization in our implants and its connection to neighboring vertebrae (Fig 6). As expected, the adjacent segment showed structures characteristic of the IVD, including highly aligned lamellae in the AF, the absence of fibers in the NP, and insertion of large collagen fibers from the AF into the vertebral body (Fig 6 and S1 Fig). Discectomized segments showed no tissue in the disc space, with no connection between collagen in the vertebral body and the intervertebral space (Fig 6 and S2 Fig). TE-IVD segments showed the presence of tissue throughout the intervertebral space. NP tissue contained some collagen, composed primarily of small, disorganized fibers (Fig 6 and S3 Fig). AF tissue was composed of large sections of organized collagen fibers that inserted in and connected to the vertebral body.

## Discussion

In the present study, we demonstrated the feasibility of total disc replacement using TE-IVDs in the canine cervical spine. Despite the challenging mechanical environment of the beagle spine, the stably implanted TE-IVDs maintained their position, integrated with the host tissue through the reorganization process, and yielded hydrated disc-like tissues over 16 weeks. Although the morphology of TE-IVD implants was distinct from the adjacent healthy IVD, implants contained an NP region with cartilaginous tissue with rounded cells and an AF region with organized fibrous tissue that inserted into the vertebral body. These implants formed organized tissue in the spine that functioned normally throughout the 16 weeks of the study.

Reconstructing a discectomized intervertebral segment with *de novo* biological disc implants is an ambitious approach to treating degenerative disc disease of the cervical spine. In 2007, the feasibility of whole allogeneic IVD transplantation was demonstrated in a clinical study with impressive results at 10 year follow up [7]. The potential clinical advantages of allogeneic IVD transplantation are limited by the availability of healthy donor discs, possible adverse immune reaction, and potential disease transmission. *De novo* TE-IVDs, the multi-compartment disc analogs using cells and biomaterials, can potentially overcome these limitations of allogeneic implants and yield favorable outcomes. A variety of *in vitro* studies and *in vivo* studies that employed animal models of nucleotomy or partial IVD resection have indicated the promise of using tissue-engineered constructs for disc replacement [9, 11, 13, 25–40], however, only three translational studies have demonstrated the in vivo efficacy of biological disc implants in the totally discectomized segment [11, 16].

In the pursuit of a biological construct that can be contained in the interbody cage to reconstruct the segment with non-bony soft tissues, Goldshlager et al. seeded mesenchymal progenitor cells into gelfoam sponges formulated with the chondrogenic agent pentosan polysulfate implanted into fully discectomized sheep lumbar segments in combination with absorbable interbody cages [41]. This study demonstrated the potential of replacing the disc segment with cartilaginous tissues to offer a means of preserving spinal motion, although the reparative tissues were distinct from those of native discs, even accompanied with ossification in some areas or specimens. Xin et al reported the aforementioned allogeneic disc transplantation and demonstrated the feasibility of human telomerase reverse transcription (hTERT) gene-tranfected NP to be incorporated with allograft IVD transplantation in the beagle cervical spine [42]. Although their results demonstrated the feasibility of allogeneic disc-based implant to be combined with genetic engineering, use of exogenous genes can hamper the clinical applicability. Previously, we developed *de novo* AF/NP composites derived from component cells to produce TE-IVDs, and demonstrated the efficacy of these discs on maintenance of disc height and disc functionality in an *in vivo* rat model [16]. The implanted TE-IVD integrated, with the host vertebrae, histologically restored the disc constitutive structures (i.e. NP and AF), and physiologically maintained NP hydration over 8 months [19].

To move this approach toward clinical application, larger animals that have a more upright cervical spine were utilized in the present study. Many breeds of small dogs, such as beagles and dachshunds, develop spontaneous cervical disc degeneration as in humans [23]. Further, previous studies have revealed that canine IVDs are exposed to stress conditions similar to or even higher than those of human discs [21–23, 43, 44]. In addition, the canine spine shows similar anatomical features and analogous degenerative processes with human discs [45]. Besides showing similar pathological changes, canines are also the only animals diagnosed and treated both medically and surgically as humans for their disc degeneration [46]. As such, canine spinal models have been previously used to investigate disc degeneration and to develop surgical treatments such as spinal fusion [47, 48] and regenerative intervention [38]. This is especially true of beagles, which are frequently used for biological approaches aimed at disc regeneration. These canines are classified as chondrodystrophic dogs due to the gradual loss of notochord cells and replacement by chondrocyte-like cells by the stage of skeletal maturity [49]. Notably the absence of such cells at skeletal maturity, which have reparative functions in the intervertebral discs, suggests that such a mechanism is not at play for implanted TE-IVD. In fact, the solely discectomized segments in the present study demonstrated no substantial repair in the disc space.

Moreover, our experiment employing stand-alone TE-IVD implantation under this clinically relevant model gave a fresh insight into development of biological disc treatment. First, mechanical stress causes instability and displacement of the implant. As evidenced by prosthetic TDR [50], displacement of implant is an arising complication when positioned in a fixation-free fashion, predominantly due to the human spine yielding severe axial loading. However, all the TE-IVDs at the C3/4 segment were stable in our study and maintained disc height up to 70% of adjacent normal discs. Biomechanics are known to be different among different levels of the cervical spine [51] and our findings may suggest biomechanics at this level of the beagle spine are more favorable to the biological disc implantation.

Our data suggested that PLL resection in the experimental segment could enhance the intraoperative stability of biological disc implants. The efficiency of PLL resection in anterior cervical decompression and fusion has been demonstrated [52]. Resecting the PLL, one of the stabilizer ligaments in the spine unit, may compromise segmental stability of the spine in the motion preserving surgery, however, Yang DL et al demonstrated that removal of the PLL improved clinical outcome of prosthetic total disc replacement via better enlargement of spinal canal without significant effect on spinal imbalance and segmental. The present study also showed that PLL resection did not pronouncedly affect the stability of treated disc segments, and to make matters more favorable, increased the stability of implanted TE-IVDs with close-to-significance.

TE-IVDs that were intraoperatively classified as stable implants, without any evidence of displacement, demonstrated better outcomes over the course of 16 weeks than the discectomy alone group, while the unstably implanted TE-IVDs demonstrated time-dependent degradation after 4 weeks. Notably, implants that were not stable displaced immediately upon removal of distraction, suggesting that additional surgical techniques maybe be needed to ensure that implants remain in place after surgery. Implants that remained in place after surgery were stable until the animals were euthanized.

In addition to the significant mechanical loading of the beagle cervical, a TE-IVD implant is also subject to a milieu with very poor nutritional supply. The indirect blood perfusion of the TE-IVD is via diffusion from blood vessels of the vertebral body through the cartilaginous endplate (EP), but there is no direct conduit of blood perfusion. In fact, the role of nutrition, critical in the long-term durability of implanted biological treatments, has been largely overlooked [53]. This is also corroborated by our finding that the NP portion of TE-IVDs at 16 weeks did not maintain as much disc height, NP hydration, and proteoglycan content as at 2 or 4 weeks. Further, axial distraction using an external fixator can enhance the regenerative capability of cell injection therapy, based on the hypothesis that individually both a distracted segment and cell injection can stimulate disc repair [54–56].

Chronic immune response to TE-IVD implants composed of allogeneic cells was notably absent in this study. Previous work has shown that transplantation of allogenic mesenchymal stem cells (MSCs) into the rat IVD invoked a minimal immune response [57], and that allogeneic articular chondrocytes transplanted into the rabbit IVD similarly caused minimal immune response [58]. These studies, combined with total disc transplantation studies conducted in beagles [42], goats [59], and humans [7], and data from the current study, collectively suggest that the disc space has a sufficient level of immunoprivilege to tolerate allogenic cell or tissue transplants. Human disc allograft was first performed in 2007 [7] and their results showed the implanted disc engrafted without any rejection, maintained disc space, and improve the patients’ symptoms. Combined with other studies, the current study corroborates that allogenic discs and disc spaces have some immune privilege as in cartilage in general. As such, the present study demonstrates that allogenic cell source is not necessary for successful outcomes, as has been noted previously [42].

Collectively, these data represent the first proof of concept studies demonstrating the regeneration of whole IVD in a large animal model. Implants engrafted successfully and persisted in the spine for up to 16 weeks, with evidence of distinct NP and AF regions. Challenges persist in maintaining function over extended periods of time and in characterizing the mechanical performance of these implants. Specifically, assessing the continued functionality of transplanted discs over long time scales (6–12 months) will be critical for establishing feasibility for human trials. Furthermore, the current study focused on transplantation of TE-IVD into healthy canine spine. The environment of the IVD in the case of degenerative disc will likely be significantly more challenging due to alterations in mechanics and diminished supply of nutrients. Future studies will assess the function of such implants in the degenerative spine to aid in the translation of this technology to clinical practice.

## Materials and methods

### Cell preparation

Cell preparation was based on previously described techniques [16, 19]. Cervical spines of skeletally mature beagles were purchased (Marshall BioResources) and IVDs were dissected out of the segments. Tissue was washed in PBS (Dulbecco’s PBS; Gibco BRL) and then separated into AF and NP regions. To isolate the component cells, tissues were dissected into small pieces and digested in 125 mL of 0.3% wt∕vol collagenase type II (Worthington Biochemicals) at 37°C for 6 h. Digested tissue was filtered through 100 μm nylon mesh (BD Biosciences) and centrifuged at 936 g for 7 min. Cells were counted and seeded in culture flasks with Ham’s F-12 media (Gibco BRL) that contained 10% fetal bovine serum (Gemini Bio Products), penicillin, (100 units∕mL), streptomycin (100 μg∕mL), amphotericin B (250 ng∕mL), and ascorbic acid (25 μg∕mL). Cells were cultured at 37°C, 5% CO_2_, and normoxia to confluence with media changes twice a week. At confluence, cells were removed from flasks with 0.05% trypsin (Gibco BRL) and counted with a hemocytometer. Cells were then seeded into TE-IVDs (Fig 1A).

### TE-IVD fabrication

T2 weighted MRI images were obtained of cervical 3/4 and 4∕5 disc levels in the beagle (imaging specifics in imaging section). The dorso-ventral and lateral-lateral dimensions of the NP region shown in the MRI were used along with the height of the disc space to model the TE-IVD core as an elliptic cylinder. A model of an injection mold for the NP was created in SolidWorks using the MRI-derived NP dimensions to guide the size of the cavities. The injection mold was then 3D printed of UV-curable watertight acrylic plastic (Shapeways). TE-IVD implant was created using established contracted collagen (AF)/alginate (NP) technique (Fig 1B) [14–16], such that the ratio of NP area to the whole contracted disc area matched the 30% ratio observed in the beagle native disc. Three percent (wt∕vol) low viscosity grade alginate (FMC BioPolymer) seeded with 25 × 10^6^ NP cells∕mL was mixed with 0.02 g∕mL CaSO_4_ (Sigma-Aldrich) to crosslink the alginate, and injected into the NP mold (Fig 1B). Cell-seeded alginate NP was then removed from molds and placed in the center of a well of a 12-well plate. Collagen type I was obtained from rat-tail tendon (Sprague Dawley, 7–8-wk old) (Pel-Freez Biologicals) using established protocols [15, 60]. One and a half milligrams per milliliter collagen gel solution seeded with 1 × 10^6^ AF cells∕mL was subsequently poured and gelled around the alginate NP. Constructs were cultured for 2 weeks in previously described media at 37°C, 5% CO_2_, and normoxia while collagen gel contracted around alginate NP to the proper AF dimensions.

### Total discectomy in canine cervical segments

Skeletally mature male beagles (n = 14) were obtained from Marshall BioResources. The animals were 12 to 18 months old at the time of surgery, with a weight of 15–25 kg. All experimental procedures were reviewed and approved by the Institutional Animal Care and Use Committee at Weill Cornell Medicine. Animals were housed in a facility accredited by the Association for the Assessment and Accreditation of Laboratory Animal Care (AAALAC) in compliance with applicable NY State, and Federal regulations. Initially, the animals underwent endotracheal intubation with administration of IV propofol, followed by a combination of inhaled isoflurane with intravenous fentanyl to maintain anesthesia. Then, the animal was placed in dorsal recumbency with the neck hyperextended and secured to the table with adhesive tape. The surgical site was prepared by clipping hair and scrubbing with chlorhexidine and betadine scrub solution. Animal was preoperatively given a dose of cefazolin 22 mg/kg IV which was repeated every 2–3 hours thereafter during the surgical procedure.

A ventral midline incision was made from the base of the larynx to the sternum. The paired sternocleidomastoideus and sternohyoideus muscles were separated with blunt dissection, exposing the trachea. Retractors were then positioned to retract the nearest carotid sheath toward the surgeon and the trachea, esophagus, and opposite carotid sheath away from the surgeon. This exposed the paired longus coli muscles, which lie on the ventral surface of the cervical vertebrae. The surgical level was identified by palpation of the prominent transverse process of C6. Small curved hemostats were used to separate the longus coli muscle overlying the ventral annulus.

The subsequent steps were carried out microscopically. After the ventral part of the AF has been incised and resected with a scalpel and a Kerrison rongeur, two self-drilling distraction pins were anteriorly inserted into the adjacent vertebrae and the disc segment was distracted with a Cervical Distractor System. Under segmental dilatation, which is augmented as the disc tissues are resected, the NP was completely extracted using a small tartar scraper, a 4–0 bone curette, and a Kerrison rongeur. The dorsal part of the AF was also resected under careful microscopic observation to expose the posterior longitudinal ligament (PLL) and confirm complete resection of the AF, and in several animals, the PLL.

One segment between C3/4 and C6/7 was totally discectomized in each animal. To determine surgical conditions that promote implant stability in a canine model, we employed different surgical levels as well as binary options between additional PLL resection or not; four of C3/4, four of C4/5, four of C5/6, and two of C6/7 segments were discectomized. Four of the animals underwent PLL resection, while the other did not. All the surgical procedures were performed by two fully-trained spine surgeons.

The neurologic examinations were performed immediately after surgery and periodically throughout the course of the study. These consisted of evaluating the animals gait in a straight line and turns to both sides, wheelbarrow on pelvic and thoracic limbs, individual limb hopping, cross extensor reflex and plantar reflex. All these tests can detect problems with coordination and strength in any of the four limbs, or injury to the spinal cord section at or caudal to the surgery site.

### In vivo total disc replacement using tissue-engineered IVD

TE-IVDs were taken out of culture and kept suspended in aforementioned media inside centrifuge tubes to maintain sterility. Constructs were brought into the operating room and implanted into the 12 segments ranging from C3/4 to C6/7 levels of the beagle spine after complete disc extraction in a single procedure, while the disc space was left untreated in the C5/6 discectomized segments of two animals. TE-IVDs were slipped down on a deliver instrument and inserted into the space. Distraction was slowly released and motion of the implanted TE-IVD was qualitatively evaluated for any motion and overall stability. Implanted TE-IVDs were categorized as displaced or stable, depending on how much of the disc remained within the intervertebral segment following release of retractors; in general, if ¼ of the disc diameter was extruded upon release we considered this displaced. Bipolar cauterization was used for homeostasis and the separated longus coli muscles were sutured to be paired with 5–0 Vicryl sutures. The wound was closed subcutaneously with 5–0 Vicryl and cutaneously with 3–0 polyamide-nylon sutures. Postoperative care was provided by Veterinary Services personnel as per the RARC veterinarian. Sutures was removed 10–14 days postoperatively. Prior to and following implantation, animals were evaluated via physical examination.

### Qualitative and quantitative magnetic resonance imaging

All the 14 animals underwent 3 Tesla MRI (Siemens Tim TRIO MRI Scanner, Erlangen, Germany) imaging at 1 month and ten at 3 months postoperatively to qualitatively monitor location and viability of implanted TE-IVDs on sagittal and axial T2-weighted images. For quantitative assessment, the voxel count and average T2 relaxation time in NP were measured according to an algorithm we previously developed [24, 61, 62]. We used a sagittal multislice multiecho pulse sequence (TR = 2000 ms, TE = 12 ms, NEX = 2, number of echoes = 12, echo spacing = 12 ms, slice thickness = 1 mm, and matrix size = 320 × 320, resolution: 125 μ m × 125 μ m × 1 mm) to create a T2 map based on fitting semi log plots of T2 signal intensity *versus* relaxation time for the 12 acquired echoes. Bruker’s proprietary program TopSpin was used for this fitting process. A color map was assigned to the resulting T2 map. Next, a standard region of interest (ROI) measuring approximately 4 mm^2^ (comprising 300 voxels) was drawn within the center NP of the healthy disc proximal to the experimental segments. The average T2-relaxation time (T2-RT) of that ROI was measured, and this value minus 3 standard deviations was used to set a subtraction threshold for all voxels in that slice. Voxels with T2 values lower than the threshold were subsequently subtracted. Thus, only voxels with T2 values representing NP tissue remained in the disc space and were then counted. At each time point, the mean voxel count of experimental segments was compared with the mean voxel count of proximal adjacent healthy discs. The mean T2-RT of NP voxels was also calculated and compared with that of the healthy adjacent control.

### Disc height measurements

X-rays were performed at one and four months to measure the disc height of treated segments. Great care was taken to achieve true lateral radiographs of the index segment. The IVD height was expressed as a disc height index, calculated by dividing disc height by adjacent vertebral body height on the basis of the modified method of Lu *et al* [63].

### Histological assessment

Animals were sacrificed one or four months postoperatively by administering a barbiturate overdose intravenously at a rate of 120 mg/kg. Spines were collected and processed for further *ex vivo* histological assessments. After fixed by 10% neutralized formalin supplemented with 1% cetylpyridinium chloride (CPC), specimens were decalcified, cut in the mid-sagittal plane, and transferred to 75% ethanol. Segments were embedded in paraffin, then cut to 5-μm thickness, and stained with Alcian Blue, Safranin-O, and Picrosirius Red.

### Data analysis and statistics

All the quantitative values from X-rays and MRIs represent the proportion of experimental to adjacent healthy control measurements, and were expressed as mean ± SD. For the analyses for continuous outcomes in disc height index, NP size, and NP hydration, generalized estimating equation (GEE) models were used to assess main effects and interaction factors of disc group and longitudinal assessment of time. Parameter estimates of means and robust standard errors are reported to describe estimated differences in mean changes from baseline controls (discectomy) across stable and displaced implantation groups for the 2, 4, 8 and 16-week time points. Statistical analysis was performed with IBM SPSS Statistics 22 (SPSS, Chicago, IL, USA). P values <0.05 were considered statistically significant.

## Supporting information

S1 Table To determine the surgical condition that promotes stable implantation, TE-IVDs were implanted in different spinal levels ranging from C3/4/ to C6/7, and with or without posterior longitudinal ligament (PLL) resection. Based on the implant stability upon distraction release, half of the TE-IVDs remained stable (n = 6), while the other half (n = 6) were considered displaced. Of note, 66.7% of the stable TE-IVDs were the ones implanted at C3/4 although the association between intraoperative implant stability and surgical level was not statistically significant (p = 0.120).
(DOCX)

S2 Table The crosstab demonstrates that the implants at C3/4 had a greater stability with a near significant association compared to the rest of the levels (p = 0.081). (DOCX)

S3 Table The segments with ligament resection all demonstrated that implant stability was nearly significantly higher than the segments with an intact posterior longitudinal ligament (p = 0.061). (DOCX)

S1 Fig Histological assessments at 16 weeks of a healthy adjacent motion segment. Brightfield images are shown for staining with Hematoxylin and eosin, Safranin O, and Picrosirius red, as well as polarized light images of Picrosirius red staining. All scale bars are 200 μm.
(TIF)

S2 Fig Histological assessments at 16 weeks of a motion segment that received a discectomy. Brightfield images are shown for staining with Hematoxylin and eosin, Safranin O, and Picrosirius red, as well as polarized light images of Picrosirius red staining. All scale bars are 200 μm.
(TIF)

S3 Fig Histological assessments at 16 weeks of a motion segment that received a TE IVD implant. Brightfield images are shown for staining with Hematoxylin and eosin, Safranin O, and Picrosirius red, as well as polarized light images of Picrosirius red staining. All scale bars are 200 μm.
(TIF)
