# Supplementary figures and images for: Total disc replacement using tissue-engineered intervertebral discs in the canine cervical spine
Source: PLoS One. 2017 Oct 20;12(10):e0185716. doi: 10.1371/journal.pone.0185716 (PMC5650136; doi:10.1371/journal.pone.0185716)

**
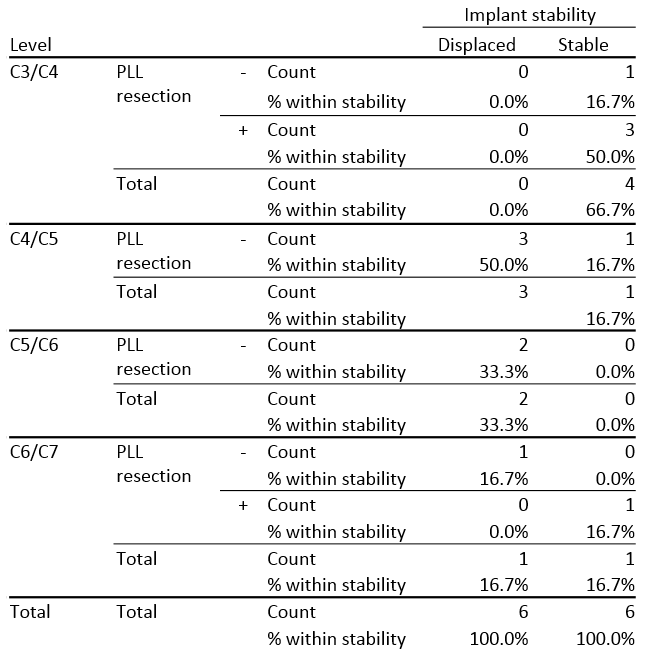
**

Supplement: S1 Table — Based on the implant stability upon distraction release, half of the TE-IVDs remained stable (n = 6), while the other half (n = 6) were considered displaced. Of note, 66.7% of the stable TE-IVDs were the ones implanted at C3/4 although the association between intraoperative implant stability and surgical level was not statistically significant (p = 0.120). (DOCX) [file pone.0185716.s001.docx]

**
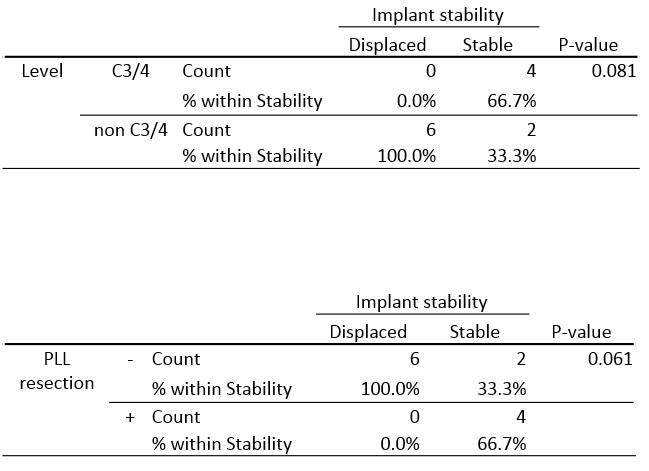
**

Supplement: S2 Table — (DOCX) [file pone.0185716.s002.docx]

**
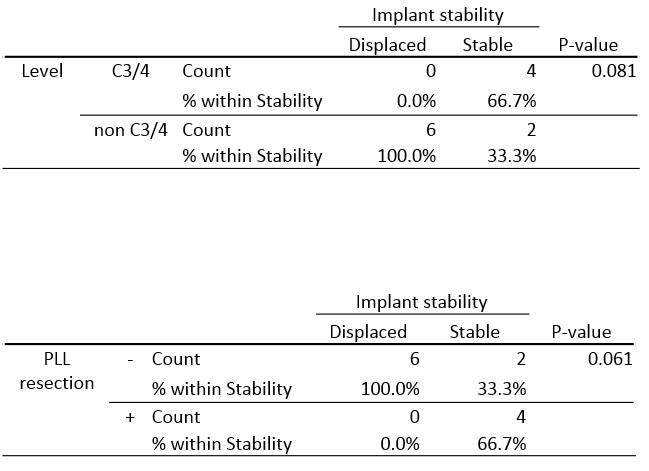
**

Supplement: S3 Table — (DOCX) [file pone.0185716.s003.docx]

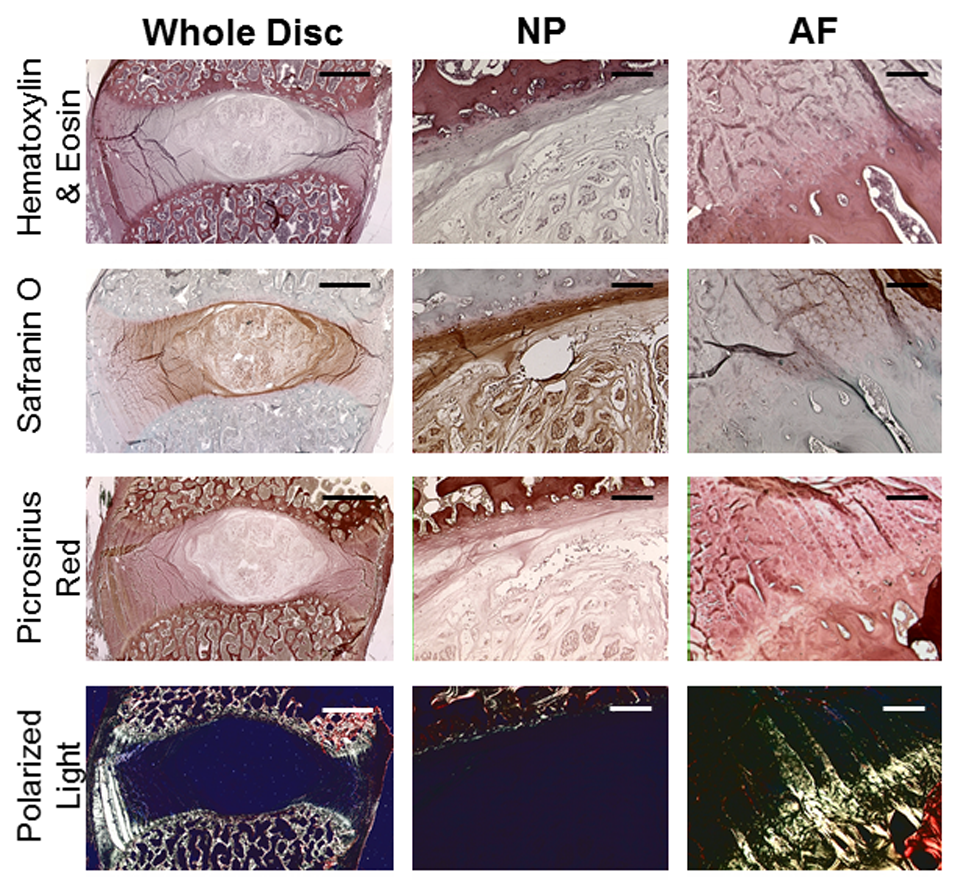

Supplement: S1 Fig — Brightfield images are shown for staining with Hematoxylin and eosin, Safranin O, and Picrosirius red, as well as polarized light images of Picrosirius red staining. All scale bars are 200 μm. (TIF) [file pone.0185716.s004.tif]

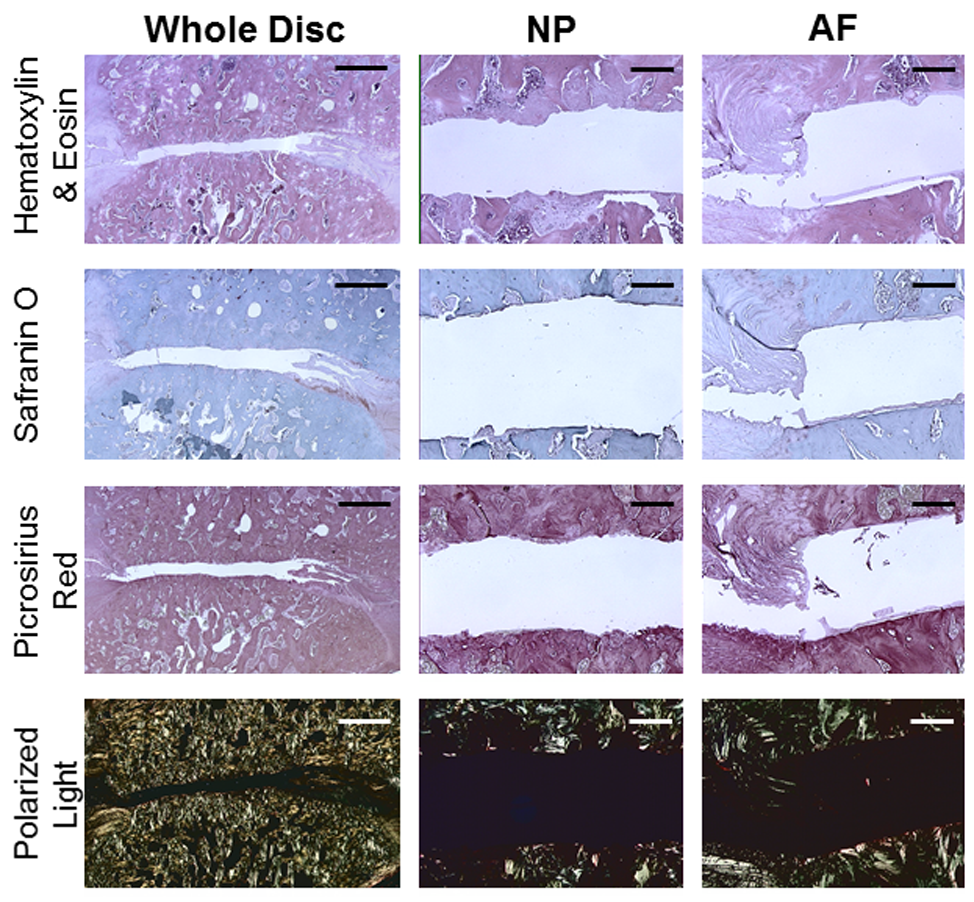

Supplement: S2 Fig — Brightfield images are shown for staining with Hematoxylin and eosin, Safranin O, and Picrosirius red, as well as polarized light images of Picrosirius red staining. All scale bars are 200 μm. (TIF) [file pone.0185716.s005.tif]

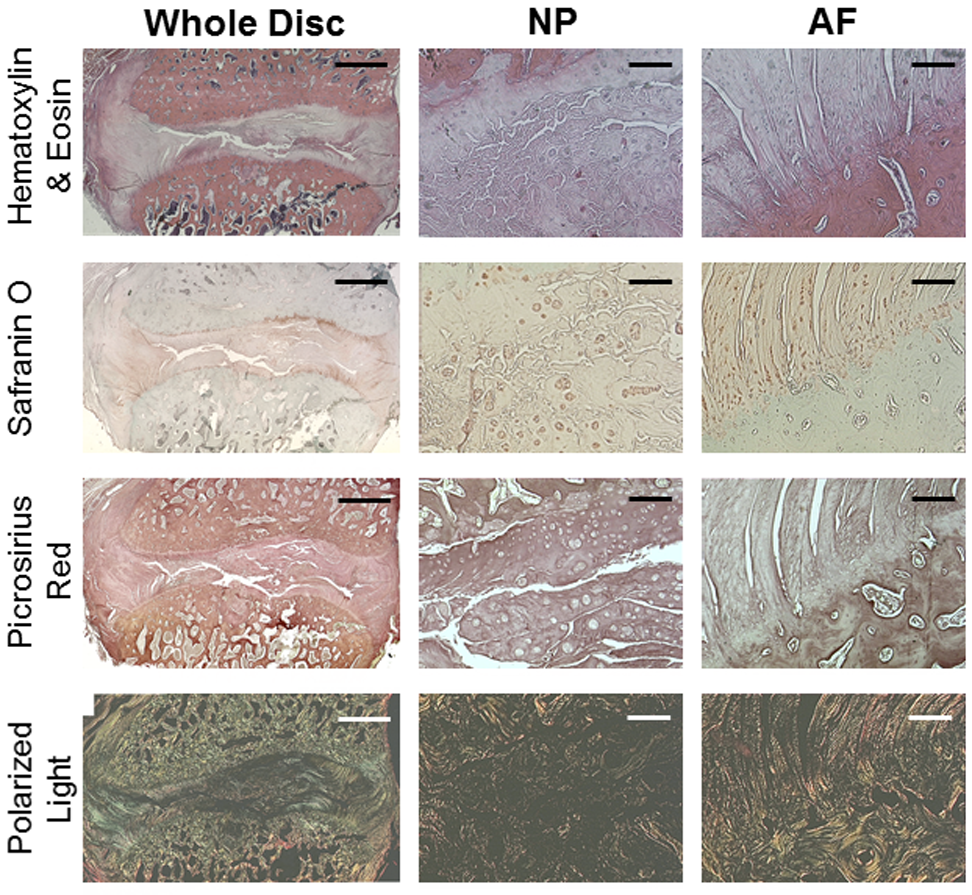

Supplement: S3 Fig — Brightfield images are shown for staining with Hematoxylin and eosin, Safranin O, and Picrosirius red, as well as polarized light images of Picrosirius red staining. All scale bars are 200 μm. (TIF) [file pone.0185716.s006.tif]
